# Medical Classes

**Published:** 1844-12

**Authors:** 


### MEDICAL CLASSES.

We have received information from several of the medical schools in the United States, and it appears that the number of students in attendance at most of them is greater than usual. The classes at the three Philadelphia schools are represented as being more numerous than they were last winter, and, altogether, it is said there are about a thousand medical students in that city. We learn, indirectly, that the schools in the city of New York are doing well. The school at Cincinnati, we are informed, has a larger class than its last, if not the largest that it has ever attracted. Nothing has reached us, in an authentic shape, from the schools of St. Louis, or that at New Orleans. Letters from Lexington mention a great falling off in the school at that place. The precise number of students does not seem to be known, but the impression in Lexington, a few days since, was that the class numbered less than 150. The Louisville Medical Institute has not only the largest class it has ever had, but the largest number of actual pupils that has at any time attended any medical school in the West. Y.

